# Two Deletion Alleles in the *C. elegans* *mir-49* gene.

**DOI:** 10.17912/micropub.biology.000236

**Published:** 2020-04-01

**Authors:** Cassandra Delich, Annabelle Dillon, Noah Winans, Shilpa Hebbar, Dustin Haskell, Anna Zinovyeva

**Affiliations:** 1 Division of Biology, Kansas State University, Manhattan, KS

**Figure 1:**
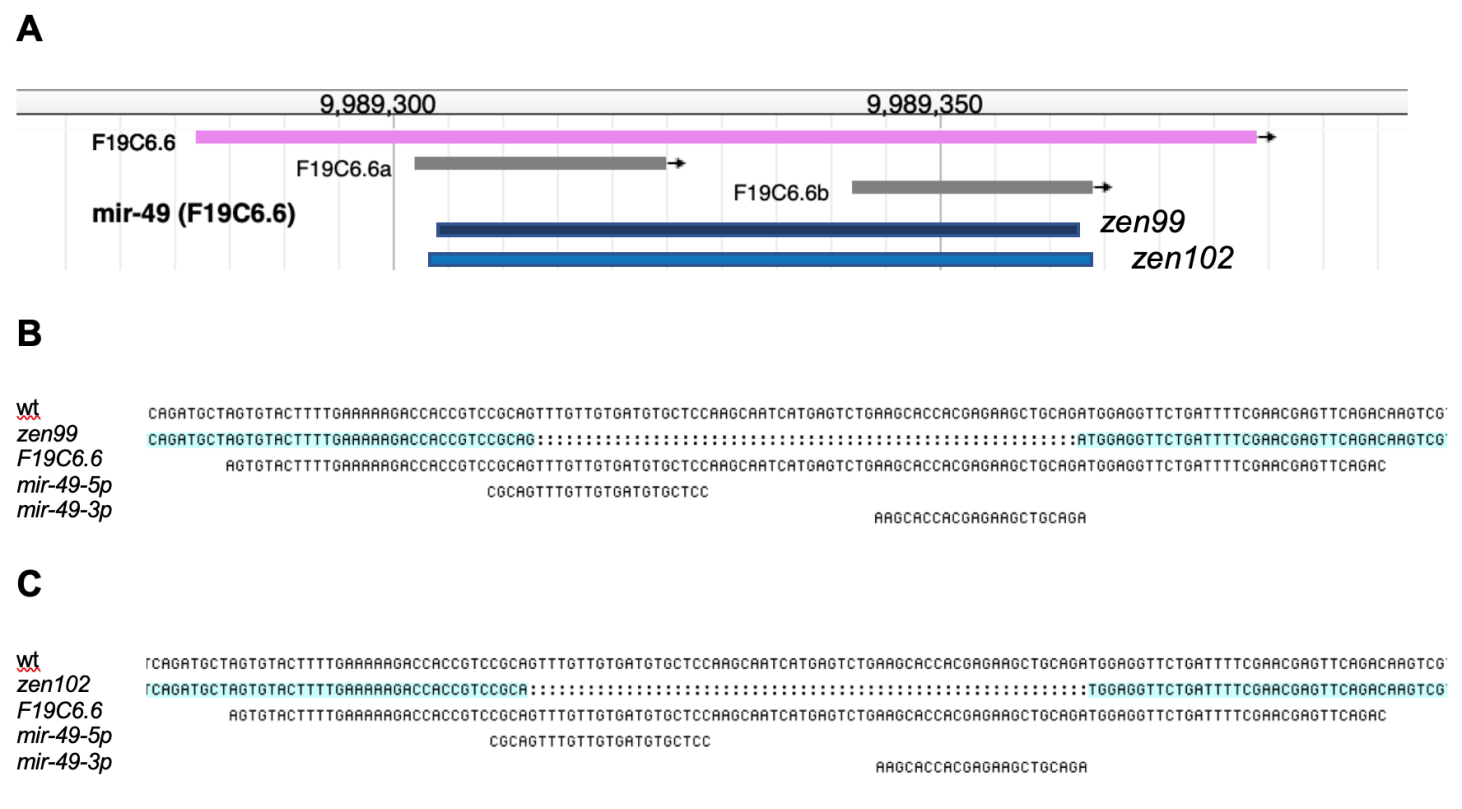
**(A)** A schematic of the *mir-49* locus and the location of the newly generated *mir-49(zen99)* and *mir-49(zen102)* deletion alleles**. (B)** *zen99* removes 56 base pairs from the *mir-49* precursor. **(C)** *zen102* removes 58 base pairs from the *mir-49* precursor.

## Description

MicroRNAs (miRNAs) are small, non-coding RNAs that post-transcriptionally repress gene expression (Gebert and MacRae, 2018). While many miRNA genes and their families have been analyzed for function (Miska *et al.* 2007, Alvarez-Saavedra and Horvitz 2010), there are microRNA genes for which loss of function alleles have not yet been generated. There are no available alleles for the *C. elegans* *mir-49* gene.

Using CRISPR-Cas9 genome editing, we generated two deletion alleles, *zen99* and *zen102*, that disrupt the *C. elegans* *mir-49* gene (Fig 1A). *mir-49(zen99)* and *mir-49(zen102)* delete 56 base pairs and 58 base pairs from the *mir-49* locus, respectively (Fig 1B and Fig 1C). Each deletion nearly completely removes both strands generated by the *mir-49* locus*, mir-49-3p* and *mir-49-5p.* Both *mir-49* alleles are homozygous viable and appear to be superficially wild type. Careful phenotypic analysis will be important to characterize the effects of the two *mir-49* deletions.

## Methods

To generate the *mir-49* deletion alleles, N2 animals were injected with the CRISPR-Cas9 components as an RNA-protein complex (Paix *et al.* 2015). The following components were used: Alt-R Cas9 (IDT, cat# 1081058) loaded with *mir-49* crRNAs (IDT, custom) (*mir-49* crRNA1 sequence: 5’-GAGCACATCACAACAAACTG-3’, *mir-49* crRNA2 sequence: 5’-GCACCACGAGAAGCTGCAGA-3’), *dpy-10* targeting guide RNA (IDT, custom) (5’-GCUACCAUAGGCACCACGAG-3’, Arribere *et al.* 2014) and tracer RNA (IDT, cat# 1072532) (AGCAUAGCAAGUUAAAAUAAGGCUAGUCCGUUAUCAACUUGAAAAAGUGGCACCGAGUCGGUGCUUU). Briefly, to load the Alt-R Cas9, the following mixture was incubated at 37^o^C for 15 minutes: 0.5µL of Alt-R Cas9, 2.4µL of tracrRNA (0.4µg/µL), 0.8µL of *mir-49* crRNA1 (0.4µg/µL), 0.8µL of *mir-49* crRNA2 (0.4µg/µL), 1.3 µL of *dpy-10* crRNA (0.1µg/µL), 1µL IDT annealing buffer (provided with Alt-R Cas9), and 3.2µL of water. Following the incubation, the mixture was spun for 2 minutes at top speed (~10,000rpm). The progeny of the injected animals was first screened for the presence of dumpy worms to identify parents positive for Cas9 activity (Arribere *et al.* 2014). F1 offspring of the Cas9-positive parents were then genotyped for the presence of potential *mir-49* deletions using the following primers: mir-49.for1 (5’-AGGCACCACCACTTACCATTCAT-3’) and mir-49.rev1 (5’-GATGACTTACAGTCGCGTCTT-3’), which generate a wild type product of ~430 bps. Independent *mir-49* deletions were identified, homozygosed, and sequenced. The resultant strains, UY264 *(mir-49(zen99))* and UY267 *(mir-49* (*zen102*)) were not outcrossed, but appear to be free of background *dpy-10* mutations. Sequencing was repeated in the next generation to ensure the stability of the generated alleles.

## Reagents

UY264 *mir-49(zen99)* and UY267 *mir-49* (*zen102*) are available upon request.
